# Evidence for glycosylation on a DNA-binding protein of *Salmonella enterica*

**DOI:** 10.1186/1475-2859-6-11

**Published:** 2007-04-02

**Authors:** Ebert S Hanna, Maria-Cristina Roque-Barreira, Emerson S Bernardes, Ademilson Panunto-Castelo, Marcelo V Sousa, Igor C Almeida, Marcelo Brocchi

**Affiliations:** 1 Departamento de Biologia Celular e Molecular e Bioagentes Patogênicos, Faculdade de Medicina de Ribeirão Preto, Universidade de São Paulo, Av. Bandeirantes 3900, Ribeirão Preto, SP 14049-900, Brazil; 2 Departamento de Enfermagem Geral e Especializada, Escola de Enfermagem de Ribeirão Preto, Universidade de São Paulo, Av. Bandeirantes 3900, Ribeirão Preto, SP 14040-902, Brazil; 3 Centro Brasileiro para Pesquisas e Serviços em Proteinas, Instituto de Biologia, Universidade de Brasília, Brasília, DF 70.910-900, Brazil; 4 Department of Biological Sciences, University of Texas at El Paso, TX 79968-0519, USA; 5 Departmento de Microbiologia e Imunologia, Instituto de Biologia, Rua Charles Darwin s/n, UNICAMP, Campinas, SP 13083-862, Brazil

## Abstract

**Background:**

All organisms living under aerobic atmosphere have powerful mechanisms that confer their macromolecules protection against oxygen reactive species. Microorganisms have developed biomolecule-protecting systems in response to starvation and/or oxidative stress, such as DNA biocrystallization with Dps (DNA-binding protein from starved cells). Dps is a protein that is produced in large amounts when the bacterial cell faces harm, which results in DNA protection. In this work, we evaluated the glycosylation in the Dps extracted from *Salmonella enterica *serovar Typhimurium. This Dps was purified from the crude extract as an 18-kDa protein, by means of affinity chromatography on an immobilized jacalin column.

**Results:**

The *N*-terminal sequencing of the jacalin-bound protein revealed 100% identity with the Dps of *S. enterica *serovar Typhimurium. Methyl-alpha-galactopyranoside inhibited the binding of Dps to jacalin in an enzyme-linked lectin assay, suggesting that the carbohydrate recognition domain (CRD) of jacalin is involved in the interaction with Dps. Furthermore, monosaccharide compositional analysis showed that Dps contained mannose, glucose, and an unknown sugar residue. Finally, jacalin-binding Dps was detected in larger amounts during the bacterial earlier growth periods, whereas high detection of total Dps was verified throughout the bacterial growth period.

**Conclusion:**

Taken together, these results indicate that Dps undergoes post-translational modifications in the pre- and early stationary phases of bacterial growth. There is also evidence that a small mannose-containing oligosaccharide is linked to this bacterial protein.

## Background

Dps was first described in *Escherichia coli *and its expression is activated when the microorganism finds itself in nutritionally limiting conditions [1] or under oxidative stress [2]. Dps is one of the major protein components in the late stationary growth phase, and both its own stability and the stability of DNA are enhanced within DNA-Dps complexes [3]. Dps proteins form dodecamers [4] and bind DNA without any apparent sequence specificity, which results in a highly ordered, multi-layered structure that physically protects DNA in an energy consumption-independent process [5]. Dps and homologous molecules have been identified in distantly related bacteria [4,6,7], suggesting that this protein plays an essential role in bacterial vitality.

Despite the limited information about the glycosylation phenomenon in prokaryotes, it is expected that their glycoproteins should share some of the structural features of eukaryotic glycoproteins. However, it is obvious that prokaryotic and eukaryotic glycoproteins should differ in terms of the biosynthetic route. As in eukaryotes, prokaryotic glycans are predominantly *O*- or *N*-linked to the protein core; nevertheless, the consensus sequences are not observed in most cases [8]. The structures of these glycans are far more diverse in prokaryotes than in eukaryotes, resembling the somatic antigen repetitive sequences of some Gram-negative bacteria in some cases [9,10]. In other cases, prokaryotic glycans display non-repetitive sequences, as in the case of the surface layer (S-layer) glycoprotein of *Clostridium thermohydrosulfuricum *[11]. They may also contain unusual carbohydrates, like the one found in *Neisseria meningitidis *pilin, where the presence of 2,4-diacetamido-2,4,6-trideoxyhexose has been detected [12,13].

Numerous functions have been attributed to the glycans of glycoproteins in eukaryotes. In prokaryotes, however, the functional characterization of glycoproteins is still unexplored, with very few exceptions. *Halobacterium halobium*, for example, seems to glycosylate the S-layer in order to maintain a rod-shaped structure [14]. Interestingly, the structure of the glycans present in the S-layer of this microorganism resembles a type of collagen. Other functions attributed to the glycan moieties in prokaryotic glycoproteins include increased stability and/or maintenance of protein conformation [15], cellular signalling and adhesion [16], physiological functions [17], and increased pathogenicity [18]. Furthermore, such glycan moieties are responsible for directing biological activity [19].

The whole process of prokaryotic glycosylation is not well understood. The general consensus is that the bacterial membrane takes part in this process, and that the mechanism involving the lipid carrier dolichol has been demonstrated [8,14,20]. It has recently been discovered that *Campylobacter jejuni *has an *N*-glycosylation system similar to that of eukaryotes, in which a group of genes named *pgl *is apparently involved [21]. The *pgl*B gene is responsible for the expression of a protein that is very similar to the Stt3p found in eukaryotes, which is an essential component of the oligosaccharyltransferase complex. Furthermore, mutation of the *pgl*A gene in *Neisseria meningitidis *suggests that it encodes a glycosyltransferase involved in the addition of a galactose residue of the trisaccharide substituent of its pilin [22]. Since the pilin of *N. meningitidis *is known to be glycosylated, it is possible that both *pgl*A and *gal*E [12] are involved in the glycosylation process.

In this study, we have purified a protein corresponding to the Dps of *S. enterica *serovar Typhimurium by affinity chromatography using a column containing immobilized jacalin. Jacalin is a lectin from *Artocarpus integrifolia *that binds galactose [23] and has high affinity for the Thomsen-Friedenreich or T-antigen disaccharide Galβ1,3GalNAc [24]. In addition, jacalin binds mannose and oligomannosides [25], which makes this lectin an important tool for evaluation of protein glycosylation. So, in this work, we present evidence that the purified Dps is glycosylated.

## Results

### J-Dps purification and staining

**Figure 1:**
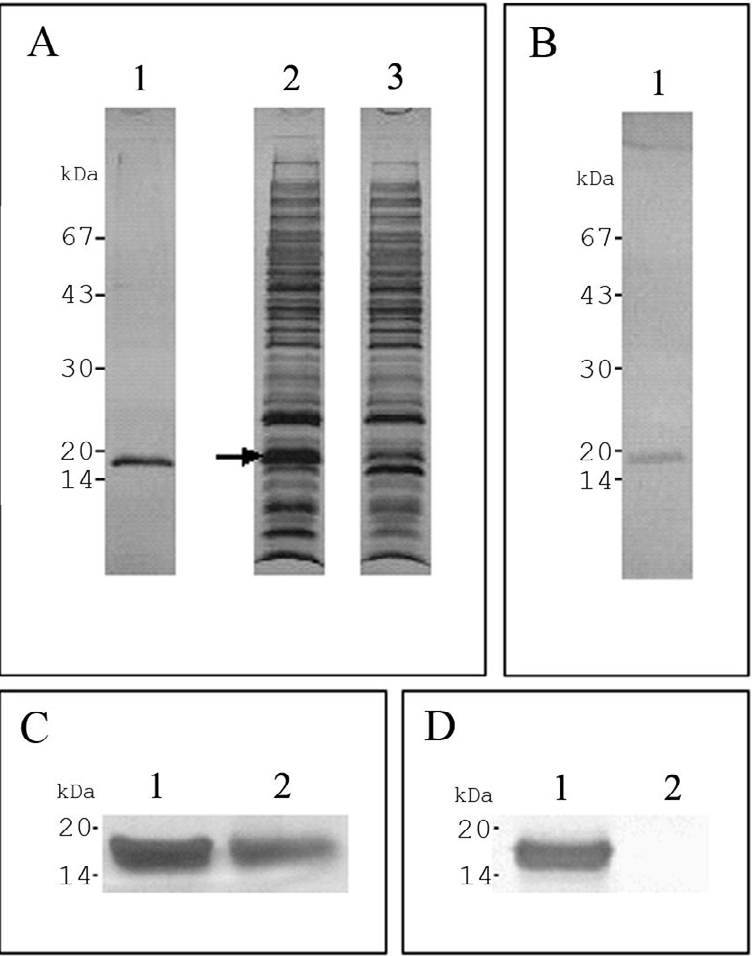
***S. enterica *has an 18-kDa jacalin binding protein**. SDS-PAGE analysis of the purified antigen of *S. enterica *stained with silver nitrate (A and C) and PAS/silver nitrate (B and D). An overnight culture of *S. enterica *Typhimurium UK-1 was pelleted, washed, and sonicated. After centrifugation, the supernatant was collected for chromatography on a jacalin column immobilized on Sepharose^®^. A single band of approximately 18 kDa delayed in the column was eluted with D-galactose and stained by both methods (lane 1, A and B). An equivalent band can be visualized (arrow) in the crude extract before chromatography (lane 2, A), and it is much less intense in the non-delayed fraction (lane 3, A). Dps was also submitted to beta-elimination reaction with NH_4_OH (C and D). Intact (native) J-Dps was stained by both methods (lane 1, C and D), whereas the NH_4_OH-treated protein was stained with silver nitrate but not with PAS (lane 2, C and D, respectively). The 18-kDa protein displayed an *N-*terminal sequence similar to that of the DNA-binding protein (Dps) of *S. enterica *Typhimurium LT-2. Molecular mass markers: lactoalbumin (14 kDa), trypsin inhibitor (20 kDa), carbonic anhydrase (30 kDa), hen egg albumin (43 kDa), and bovine serum albumin (67 kDa).

One liter of LB-cultured *S. enterica *produced approximately 1.0 g of a soluble crude extract whose protein electrophoretic profile is shown in Fig. 1A (lane 2). The jacalin-bound fraction of the crude extract was eluted from the affinity column with 0.2 M D-galactose-containing buffer. This chromatographic procedure allowed purification of an 18-kDa glycoprotein, which was stained as protein by the silver method and as glycoconjugate by the PAS method (lane 1, fig. 1A and 1B, respectively). Because the *N*-terminal sequence (STAKLVKTKASNLLYTRNDV) of this protein, obtained by Edman degradation, showed 100% identity to the DNA-binding protein Dps of *S. enterica *Typhimurium LT-2 the jacalin-bound preparation was denoted J-Dps. A band with apparent molecular mass and staining properties similar to those of Dps was clearly visualized in the *S. enterica *crude extract (Fig. 1A, lane 2), but barely seen in the unbound fraction of the crude extract (Fig. 1A, lane 3). In addition, when J-Dps was treated to undergo beta-elimination of *O*-glycans, it remained silver-stained (Fig. 1C, lane 2) but it was not possible to stain it with PAS (Fig. 1D, lane 2). These observations suggest that *O*-linked carbohydrate is present in the J-Dps structure.

### Lectin Binding to J-Dps

**Figure 2:**
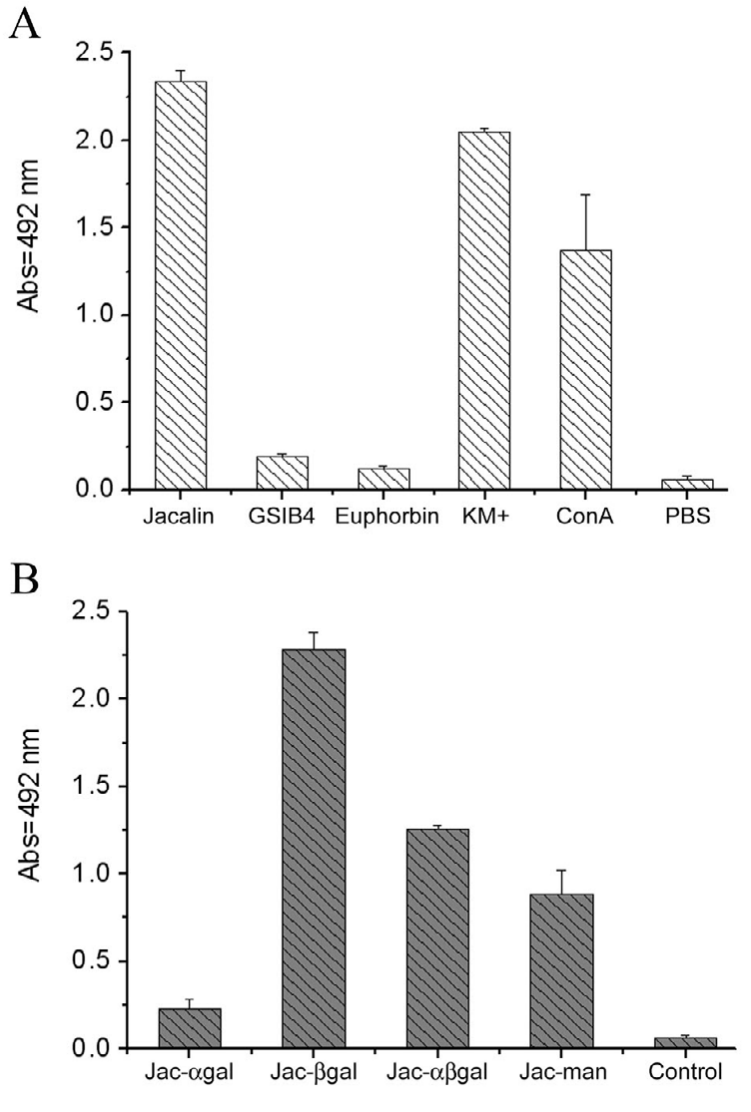
**Dps binds to jacalin, via CRD, but not to other galactose-binding lectins**. Isolectin B4 of *Griffonia simplicifolia *(GSIB4), euphorbin from *Euphorbia milli*, jacalin and KM+ from *Artocarpus integrifolia*, and concanavalin-A were biotinylated and their interactions with Dps in solid phase were assessed (A). The negative control was performed in the absence of lectins. The binding of biotinylated jacalin to Dps in solid phase was assessed in the presence of sugars at a concentration of 0.1 M (B). The alpha-anomer of methyl-galactose (Jac-αgal) inhibited the jacalin/Dps binding drastically, whereas the beta anomer (Jac-βgal) did not. A solution of D-galactose (Jac-αβgal) and D-mannose (Jac-man) partially inhibited this interaction. PBS was the control in the absence of sugars and lectins.

The jacalin carbohydrate-binding specificity has been reported on the basis of its specific interaction with the T-antigen disaccharide Galβ1,3GalNAc [24], but a recent investigation has led to the suggestion that there is promiscuity in its sugar specificity [26]. Knowing that lectin-binding contributes to the characterization of glycoproteins, we re-examined the lectin-binding ability of J-Dps by means of a microplate lectin-binding assay. Besides jacalin, other biotinylated lectins such as GSIB4 (specific for α-galactosides), euphorbin (specific for *N*-acetyl-galactosamine), and KM+ and concanavalin-A (both specific for mannose-containing glycans) were probed. Among the galactose-binding lectins, only jacalin was able to bind J-Dps, providing optical density readings 20-fold higher than those provided by GSIB4 or euphorbin (Fig. 2A). Both mannose-binding lectins bound to J-Dps, although their affinity for J-Dps was lower than that of jacalin. The sugar recognition dependence of the jacalin binding to J-Dps was confirmed by means of an inhibition assay in which the alpha-anomer of methyl-D-galactopyranoside (Me-αGal *p*) inhibited the jacalin/Dps interaction, whereas the beta-anomer (Me-βGal *p*) did not (Fig. 2B). We also showed that D-galactose and D-mannose partially inhibited J-Dps recognition. Because jacalin binding to J-Dps was strongly inhibited by Me-αGal *p *and inhibited by D-mannose, we hypothesize that the carbohydrate recognition domain (CRD) of the lectin is involved in such a binding.

### Mass Spectrometry Analysis of J-Dps

**Figure 3:**
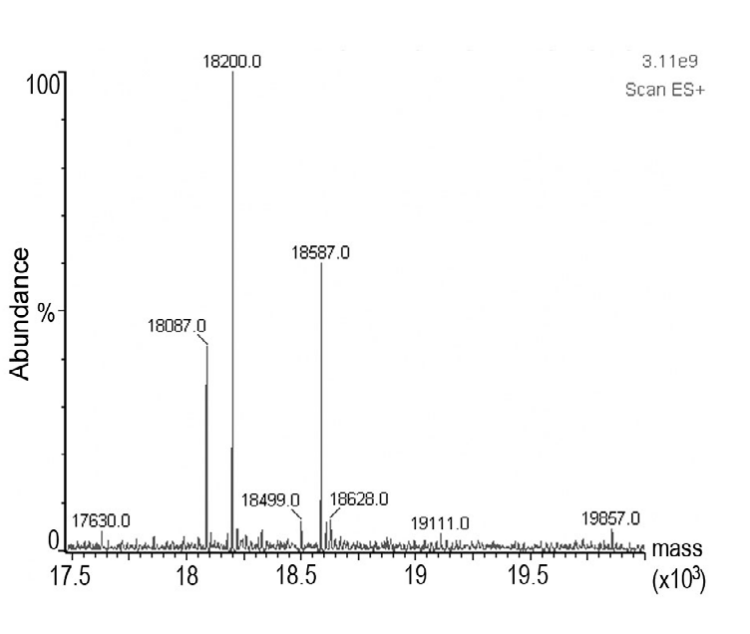
**Molecular mass analysis of Dps by ESI-MS**. J-Dps was analyzed by ESI-MS (positive-ion mode). The peaks represent the average molecular mass of multi-charged species after mass deconvolution.

Analysis of the J-Dps preparation by ESI-MS, solubilized in formic acid 10%, showed a collection of peaks with variable molecular masses (Fig. 3). The sequence of Dps protein predicted for *Salmonella*, with excised *N*-terminal methionine, has a deduced molecular mass of 18,586 Da, which is very close (18,587 Da) to the molecular mass of the second most abundant peak. We could also detect varying amounts of degradation products resulting from the loss of the *N*-terminal serine (18,499 Da) up to lysine (18,200 Da) and leucine (18,087 Da). Other minor peaks could also be detected and their correlation with the Dps molecule remains to be investigated.

**Figure 4:**
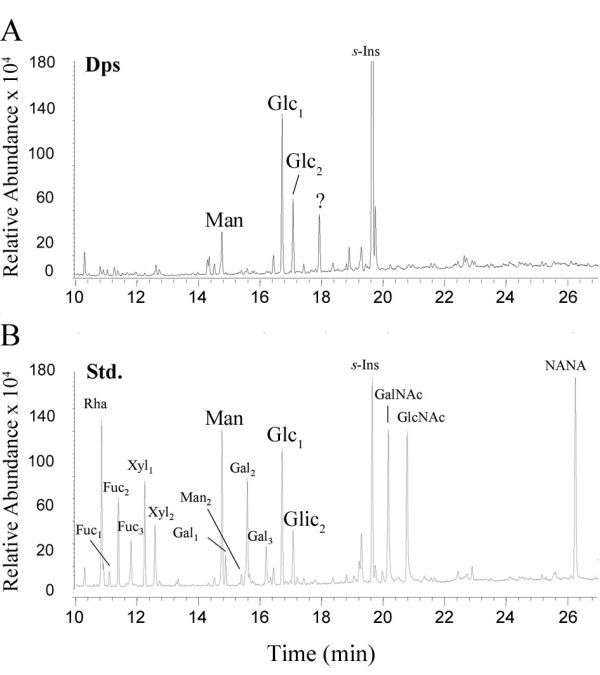
**Total carbohydrate analysis of Dps by GC-MS**. J-Dps (200 picomoles) was hydrolyzed with methanolic 0.5 N HCl for 4h, at 85°C, re-N-acetylated and analyzed by GC-MS as trimethylsilyl (TMS) derivatives. s-Ins, scyllo-inositol (internal standard, 1 nmol); Std., carbohydrate standard mixture containing 500 pmol of each of the sugars Rha, Fuc, Xyl, Man, Gal, and Glc, and 1 nmol of each of the sugars GalNAc, GlcNAc, and NeuAc (NANA). Subscript numbers indicate Fuc, Xyl, Man, Gal, and Glc isomers. The 1-O-methyl-Manp-TMS4 derivative in the Dps sample was confirmed after electron impact fragmentation. Characteristic ions at *m/z *73, 133, 147, 204, and 128, similar in intensity to those obtained from the fragmentation of an authentic 1-O-methyl-Man *p*-TMS4 standard, were observed. The unknown ion species (?) could not be identified through database matching of the electron impact fragmentation spectrum.

Monosaccharide composition as determined by GC-MS analysis of the trimethylsilyl (TMS) derivatives indicated the presence of glucose, mannose, and an unknown component in significant amount (Fig. 4). Because glucose has commonly been associated with sample contamination, we focused our attention on the presence of both mannose and the unknown component only. In spite of having been purified on a jacalin column, we could not identify any traces of galactose or *N*-acetyl-galactosamine in the J-Dps sample.

### Glycosylated Dps is produced in the pre- and early stationary phases of bacterial growth

**Figure 5:**
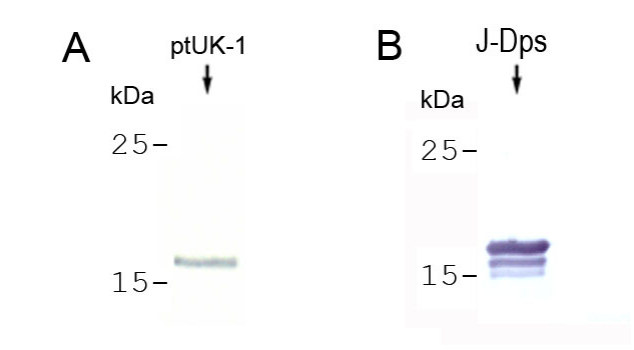
**Western blot of *S. enterica *antigens and J-Dps detection with anti-Dps**. A solution of the cell-lysed constituents (A) or the jacalin column preparation (B) was fractionated by SDS-PAGE and blotted onto nitrocellulose paper. In A, a single constituent of approximately 18 kDa (arrow) was detected by the antibody anti-Dps, among several antigens of *S. enterica *Typhimurium UK-1 (ptUK-1). In B, a pattern of triplet bands is observed in the J-Dps preparation. Molecular mass markers (Rainbow^®^, Amersham Biosciences, GE Healthcare, Piscataway, NJ, USA) are indicated on the left.

Antibodies anti-Dps, produced by immunization of BALB/c mice, were highly reactive (up to 1:128,000 serum dilution) with the antigen-coated microplates (data not shown), and they only recognized the 18-kDa antigen in the immunoblotting analysis of an *S. enterica *cell-lysed culture (Fig. 5A). In the Western blot of the J-Dps preparation, narrow doublet and triplet bands are revealed (Fig. 5B, J-Dps), ranging from 15-kDa to18 kDa. This same pattern of bands has been observed in recombinant Dps over-expressed in *S. enterica *cells (unpublished results).

**Figure 6:**
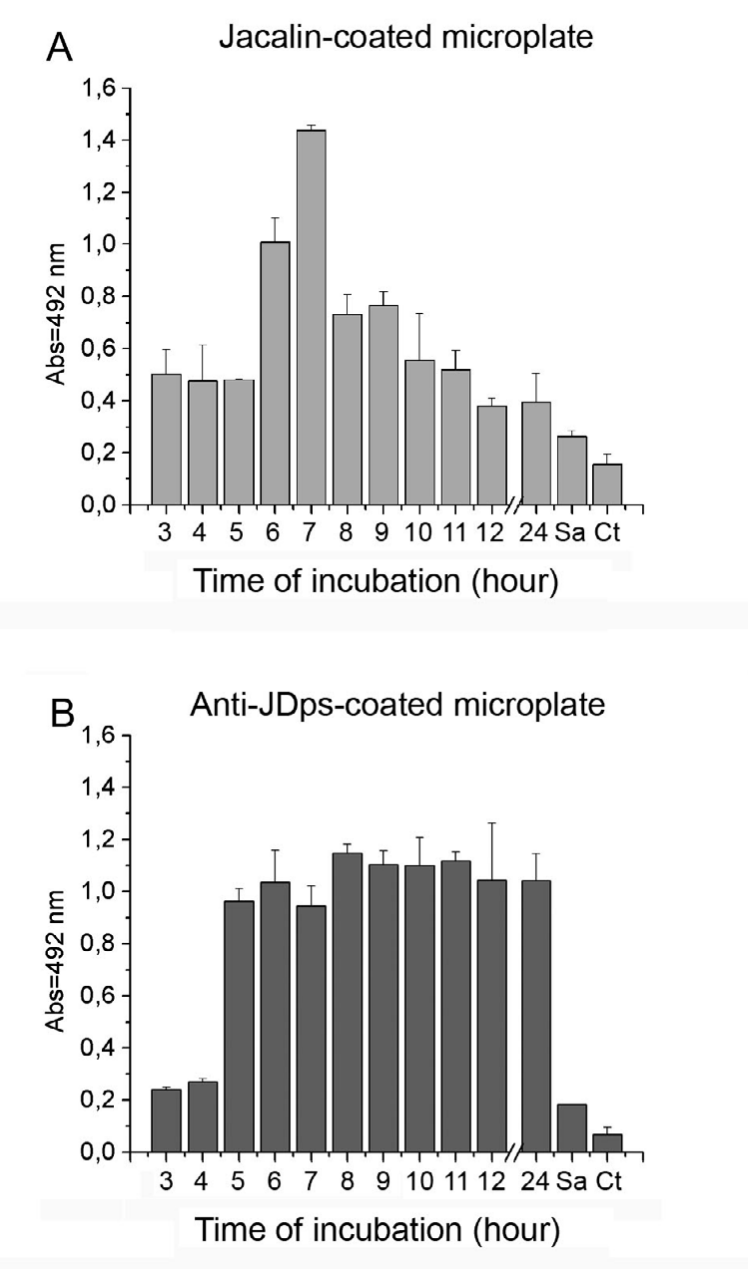
**Detection of Dps and J-Dps during the growth phase of *S. enterica***. Crude extracts of *S. enterica *(A: 25 μg and B: 5 μg each sample) from different growth periods were added (100 μl/well) to the wells of a microplate coated with jacalin (A) or rabbit antibody anti-Dps (B). Dps detection was performed, in both cases, with murine antibody anti-Dps. Results are the read average and standard deviation of triplicates, and are expressed as the absorbance at 492 nm. **Sa**, crude extract of *Staphylococcus aureus*; **Ctrl**, control with no added crude extract.

Anti-Dps was used to estimate the relative antigen concentration during the *S. enterica *growth phase. Cell-lysate extracts obtained from bacteria cultured for different time intervals were added to jacalin- or anti-Dps-coated wells of a microplate. Binding was detected through the addition of anti-Dps antibody and revealed by labeled anti-murine IgG. Dps capturing by specific antibodies confirmed literature data concerning the higher antigen concentration in the late stationary phase of bacterial growth (Fig. 6A). On the other hand, the relative concentration of Dps captured by jacalin was higher in the earlier growth stages (from 5 to 7 hours), decreasing thereafter (from 8 to 24 hours). Maximum Dps concentration was captured by jacalin coating in the lysate of cells taken from 7-hour cultures (Fig. 6B).

## Discussion

Dps expression has been proven to be controlled by either the Sigma factor δ^S ^in the stationary phase of bacterial growth or the transcriptional activator *oxy*R when the bacterial cell faces oxidative hazards [27]. *In vitro *growth results in depletion of all the nutrients, leading to morphological and physiological changes in the bacterial cells that will help them adapt to an environment where carbon sources are scarce. In this way, bacterial cells in the stationary growth phase are smaller, both the protein synthesis and the number of ribosomes are reduced, and the DNA molecules are sequestered and structurally protected by energy-independent intracellular assemblies [3]. The observation that an 18-kDa protein of *S. enterica *was able to bind to immobilized jacalin and the fact that this protein has an *N*-terminal sequence displaying 100% identity to Dps have led us to hypothesize that glycosylation occurs in the DNA-binding protein of this bacterium strain. In addition, SDS-PAGE analysis of J-Dps stained for proteins and glycoconjugates reinforces the glycosylation hypothesis. It is important to bear in mind some aspects of these staining methods: i) proteins are stained with silver nitrate, whereas carbohydrates in glycoconjugates are oxidized by periodate and stained by silver nitrate dissolved in alkaline buffer (PAS-silver nitrate); ii) PAS-silver nitrate is likely to stain proteins, but with lower sensitivity; iii) in the case of glycoproteins, silver nitrate might have a double function, thus staining oligosaccharides and polypeptides; iv) in our assays we verified that the glycoprotein hen-egg albumin is stained by PAS-silver nitrate, whereas the non-glycosylated protein bovine albumin is not (data not shown). Although Dps has already been well characterized in *Enterobacteriaceae *as well as in *Salmonella *[1,4,28], there is no report of glycosylation on its molecule. In other words, Dps purification by affinity chromatography on lectin-immobilized columns has never been considered.

During beta elimination, the beta-carbon of the amino acid residue(s) to which the glycan(s) was/were attached react(s) with ammonium hydroxide to yield a modified amino acid residue and release the *O*-linked oligosaccharide(s). The beta elimination reaction shown in Fig. 1 made the detection of J-Dps by the PAS-silver nitrate staining method impossible, corroborating the discriminatory capacity of this method. This result supports the idea of glycosylation in the Dps molecule.

We have investigated sugar inhibition of jacalin binding and our results are in agreement with the jacalin binding specificity [29]. The fact that the binding of biotinylated jacalin to coated J-Dps was totally abolished by Me-αGal *p*, and not by the Me-βGal *p*, indicates that the carbohydrate recognition domain (CRD) of the lectin is involved in this process. This result has led us to another clue with respect to the Dps glycosylation purpose.

Among the lectins tested herein, jacalin has remarkable ability to interact with J-Dps. Because jacalin is mainly known as a galactoside-binding lectin, we conjectured that J-Dps could contain galactoside residues on its molecule. However, other tested galactose-binding lectins did not recognize J-Dps, while mannose-binding lectins, such as ConA and KM+, did interact with J-Dps (Fig. 2A). A 0.2 M mannose solution inhibited the J-Dps interaction with jacalin (Fig. 2B), ConA, and KM+ (not shown). As expected, Me-αGal *p*, a higher affinity ligand for jacalin, inhibited jacalin binding to J-Dps, a fact that was attributed to the hindrance of the jacalin CRD. Interpretation of the results concerning J-Dps binding to lectins has become clear from the results obtained with the carbohydrate analysis by GC-MS of the J-Dps molecule. Carbohydrate compositional analysis gave evidence of the presence of mannose, glucose, and a third unknown residue. Because glucose has been commonly identified as contaminant species in this kind of assay, we have considered that its presence is irrelevant. Indeed, D-mannose, and not D-galactose, was detected, leading us to assume that binding to jacalin is attributed to a secondary affinity of the lectin for mannose and oligomannosides [25] associated to the unknown residue, which might have some role in this binding.

Furthermore, unusual saccharide residues have been reported as components of many glycoproteins or polysaccharides in prokaryotes [30-33], and this might be the case of the unknown residue that did not match any component of the standard. The detailed structure of the J-Dps oligosaccharide by meticulous structural analysis using GC-MS, ESI-MS, nuclear magnetic resonance (NMR), and other analytical techniques is underway.

Some points concerning characteristics of the Dps molecule are important and should be mentioned, with respect to the ESI-MS analysis. Firstly, self-aggregation of *E. coli *Dps is related to the properties of the amino terminus of this protein [34]. In fact, self-aggregation of this protein leads to the formation of oligomers of variable size, which tend to precipitate. This might explain why we had to solubilize J-Dps in formic acid 10 % to properly analyze it by ESI-MS. Secondly, Dps is supposed to lose up to 6 residues at the *N*-terminus [1], producing varying amounts of a minor degradation product. In fact, we were able to detect these *N*-terminus degradation specimens in different J-Dps preparations. Finally, the two most potential *O*-glycosylation sites as determined by NetOGlyc 3.1 [35] lie close to near threshold values for this algorithm and are located on positions 2 and 3 of the polypeptide chain. These positions are supposed to be excised away by the degradation predicted at the *N*-terminus. As in eukaryotes [36], nuclear protein glycosylation can be very labile, and J-Dps solubilization could have accelerated deglycosylation and/or *N*-terminus degradation processes, making the detection of any glycoprotein traces difficult. J-Dps preparation after long term storage at -20°C showed a pattern of doublet/triplet bands. A similar pattern is observed with the fresh preparations of Dps obtained from recombinant strains (unpublished results). However, we could not establish any correlation between the glycosylation and degradation processes.

In eukaryotes, there are many transcriptional factors that, in certain periods of time, interchange the phosphorylation/*O*-GlcNAc-glycosylation state to exert stress-protective functions for important biomolecules such as DNA. Nuclear proteins containing *O*-GlcNAc have been shown to form reversible multimers and to be phosphorylated [37,38]. Addition of *O*-GlcNAc to the protein backbone is dynamic and it has been shown to respond to cellular stress and changes in glucose metabolism [36]. Interestingly, Dps was identified as a phosphoprotein in a model study of *E. coli *physiology [39]. The present work indicates that *S. enterica *Dps is a glycoprotein, which supposedly adds important data to a phenomenon that has been proven to be ubiquitous in eukaryotes.

Several works have demonstrated that the bacterial cell accumulates large amounts of Dps during the stationary growth phase [1,3,5,27,28,40]. Thus, when the bacterial cell faces nutritional depletion during the stationary phase, Dps rises up to 23–25-fold its initial amount per cell, thus becoming the most abundant component in the cell during that period [41]. Likewise, Dps concentration is also supposed to be regulated by a proteolysis mechanism, in which the enzymes ClpXP and ClpAP might play an important role in the modulation of Dps degradation [42]. We have observed that the jacalin-coated microplate captured more Dps from the crude extract of *S. enterica *obtained from earlier growth periods than from the crude extract obtained from later growth phases. This reveals a relative concentration curve opposed to the one reported for Dps concentration [41], which was confirmed herein through the assay using anti-Dps coated microplates. Once Dps production and accumulation is primarily associated with stationary phase, it would be possible that another immuno-reactive product in logarithmically growing cells could be cross-reacting with the anti-Dps antibody. However, the monospecificity of the anti-Dps antibodies produced in BALB/c mice is suggested by the development of a single band in the immunoblot of crude *S. enterica *lysate. In addition, detection of Dps in cells growing logarithmically is consistent with a previous systematic immunoblot analysis of *E. coli *cell lysates, showing that in the exponential growth phase one single cell contains around 6,000 Dps molecules [41]. Therefore, we are tempted to suggest that the *S. enterica *component captured by jacalin on each well was most probably Dps, and there might be a kind of modulation that leads to the acquisition of suitable glycosylated/non-glycosylated molecule ratios in the bacterial cell in certain growth periods. Because Dps is a DNA-binding protein and its binding is associated with nutritional depletion or oxidative stress, glycosylation appears to be inversely related to the DNA-binding process. Thus, bacterial cells growing exponentially should keep a certain amount of Dps for an eventual stress condition where, instead of the *de novo *synthesis, pre-existing molecules would be ready to bind and protect DNA, and glycosylation might play a role in this mechanism.

## Conclusion

It is clear that Dps production is finely regulated by the bacterial cell, and its production responds to many stimuli in the growth medium. Our work has shown that immobilized jacalin hooked one of the several constituents of the crude extract of *S. enterica*. Whether glycosylation really occurs in any other bacterial DNA-binding protein is an intriguing question. Dps is a stress inducible DNA binding protein, and we have gathered evidence that this molecule is, at least for a certain period of the bacterial cell physiology, glycosylated. To our knowledge, this is the first description of glycosylation in a prokaryotic DNA-binding protein.

## Methods

### Bacterial strain, growth and processing

The strain UK-1 (Universal Killer) of *Salmonella enterica *serovar Typhimurium χ3761 was kindly provided by Dr. Roy Curtis III (Washington University, Washington, DC, USA), and cells were grown in Luria-Bertani medium at 37°C for 18 h at 200 rpm. Cells were harvested by centrifugation at 2,300 *g *(10 min, 4°C), and the pellet was washed twice with phosphate buffer-saline containing sodium azide 0.2% (PBS-A). Bacterial lysis was performed by sonication (3 pulses, 30 seconds each), followed by centrifugation at 17,800 *g*, (15 min, 4°C), to obtain the crude extract of *S. enterica *in the supernatant. Crude extracts of recombinant strains were firstly cleared by FPLC before purification by affinity chromatography (unpublished results). Protein concentration was measured by the bicinchoninic acid (BCA) assay (Pierce Chemical Co., Rockford, IL., USA).

### Purification and analysis of the 18-kDa *S. enterica *antigen

The jacalin column was obtained as described previously [23] and loaded with the crude extract of *S. enterica*. The column was then washed with 10 column volumes of PBS-A, and the adsorbed fraction was eluted in a sharp peak with 0.2 M D-galactose in PBS-A. The column eluate was monitored by UV absorbance at 280 nm, dialyzed against water in a YM-10 membrane (Amicon Division, W.R. Grace & Co., Beverly, USA), and examined by SDS-PAGE 12.5% in Mini V-8.10 Vertical gel Electrophoresis System (Gibco BRL, Gaithersburg, MD, USA). Gel was stained with either silver nitrate for proteins [43] or periodic acid Schiff (PAS) for glycoconjugates [44]. *N*-terminal sequencing was performed on a protein sequencing system (model 477A-120A, Applied Biosystems, Inc., Foster City, CA, USA) with some hardware and software modifications [45], following transfer from SDS gels to ProBlot PVDF membrane (Applied Biosystems, Inc.), as described by LeGendre and Matsudaira [46]. The *N*-terminal sequence was used to search for similar sequences in the genomic bank of *S. enterica *serovar Typhimurium, in the site of the Washington University School of Medicine (St. Louis, MO, USA), using the Blast 2.0 local program. The first 20 amino acids were identical to the *N*-terminus of the Dps protein from *S. enterica *serovar Typhimurium LT-2, and this fraction was designated J-Dps (J stands for jacalin-purified).

Beta-elimination of glycans from glycopeptides with ammonium hydroxide (NH_4_OH) was performed as described by Rademaker and co-workers [47].

### Enzyme-linked lectin assays (ELLA)

Jacalin and KM+, both extracted from the seeds of *Artocarpus integrifolia *[23,48]; isolectin B-4, extracted from the leaves of *Griffonia simplicifolia *[49]; euphorbin, obtained from the latex of *Euphorbia milii *var. *milii *[50]; and concanavalin-A (Sigma, St. Quentin Fallavier, France) were biotinylated with the EZ-Link Sulfo-NHS Biotin Kit, according to the manufacturer's instructions (Pierce Chemical Co.). Each well of a polystyrene microtiter plate (96-well plate, Nunc, Inc., Naperville, IL, USA) was coated with one microgram of J-Dps diluted in 50 mM carbonate-bicarbonate buffer (50 μl/well), and incubated for 18 h at 4°C. Wells were washed with PBS containing 0.05% Tween-20 (PBS-T). Unspecific reactions were blocked using 100 μl/well of 1% gelatin in PBS-T (blocking buffer), followed by incubation for 1 h at 37°C. The plate was washed once with PBS-T and incubated with 100 μl/well of biotinylated lectins in blocking buffer for 2 h at 37°C. After washing with PBS-T, the plate was incubated with 100 μl/well of neutravidin-peroxidase (Sigma Chemical Co., St. Louis, MO, USA), diluted 1:2000 in blocking buffer, for 1 h at 37°C). After washing with PBS-T, 100 μl of the substrate buffer containing 19 μl 0.1% hydrogen peroxide, 5 mg orthophenylenediamine (OPD), 3 ml 0.1 M citric acid, 3.2 ml 0.2 M sodium phosphate, and 6.25 ml deionized water were added to each well. The reaction was quenched with 50 μl 2 M sulphuric acid, and the absorbance was read using a Multiskan microplate reader (MMC/340P, version 2.20, Labsystems, Helsinki, Finland), at 492 nm.

The sugar inhibition assay was performed as described above, including an extra step in the carbohydrate inhibition test. Biotinylated jacalin was incubated with 50 mM alpha-methyl-D-galactose, 50 mM beta-methyl-D-galactose, 50 mM D-galactose, or 50 mM D-mannose prior to adhesion to J-Dps in the microtiter plate. The negative control was the lectin-absent assay.

### Immunoblot

BALB/c mice and rabbits were used for production of anti-Dps antibodies. Immunization was carried out by intraperitoneal injection of 10 μg (mice) or 100 μg (rabbit) of J-Dps in PBS, emulsified in complete Freund's adjuvant, followed by three boosts (on days 21, 28, and 35) with the same amount of J-Dps, emulsified in the incomplete Freund's adjuvant. The antiserum was obtained 1 week after the last boost and its reactivity with Dps was analyzed by immunoblotting. Pellets from 1.5 ml culture of *S. enterica *were lysed in 100 μl reducing buffer [recipe for 10 ml: 3.55 ml deionized water, 1.25 ml 0.5 M Tris-HCl (pH 6.8), 2.5 ml glycerol, 2.0 ml sodium dodecyl sulfate 10% (w/v), 0.2 ml bromophenol blue 0.5% (w/v), and 0.5 ml 2-mercapto-ethanol)]. Polyacrylamide gel was electrotransferred to the nitrocellulose membrane (Millipore Co., Billerica, MA, USA). Nonspecific interactions were blocked with 3% skimmed milk (Nestlé Brazil) in PBS-T (see above) for 2 h at 25°C. Dps was detected by incubation overnight with anti-Dps serum diluted at 1:4000 in 1% skimmed milk in PBS-T. The antigen-antibody reaction was detected after overnight incubation with peroxidase conjugate anti-mouse or anti-rabbit IgG antibody (Sigma Chemical Co.) diluted 1:2000 in 1% skimmed milk in PBS-T, and revealed with 0.3% 4-chloro-naphtol [20 ml 0.05 M Tris-Cl (pH 6.8), 7 μl hydrogen peroxide, and 5 ml 4-chloro-naphtol 0.3% (w/v in methanol)].

### ELISA

Crude extracts of *S. enterica *from different growth periods were diluted to equalize the amount of protein with that of the less concentrated sample. Rabbit anti-Dps (1 μg/well) or purified jacalin (1 μg/well) were coated on microtiter plates. The jacalin-coated plate was incubated for 2 h at 37°C with 25 μg (100 μl/well) of one of the samples of the crude extract (3–18 and 24 hours of growth), whereas the anti-Dps-coated plate was submitted to the same incubation conditions with 5 μg (100 μl/well) of the samples. Murine anti-Dps (100 μl/well) diluted 1:4000 was used as primary antibody, and rabbit peroxidase-conjugated anti-mouse IgG antibody (100 μl/well) diluted 1:2000 (Sigma Chemical Co.) was the secondary antibody. The crude extract from an overnight culture of *Staphylococcus aureus *was used as control. All buffers used for coating, blocking, washing, substrate reaction, and reading were the same as described above. Results are expressed as the absorbance at 492 nm.

### Molecular mass analysis

Protein molecular mass profiles were determined by electrospray ionization mass spectrometry (ESI-MS) using a Finnigan LCQ Duo ion-trap mass spectrometer (Thermo Electron Co., San Jose, CA, USA). Spectra were acquired in the positive-ion mode, and molecular masses were calculated by mass deconvolution using the Xcalibur software (Thermo Electron Co.). Potential glycosylation sites were predicted using GlycoMod and NetOGlyc 3.1 [51].

### Carbohydrate analysis

An aliquot of J-Dps (200 picomoles) was hydrolyzed with 0.5 M hydrochloric acid-methanol (Supelco, Bellefonte, PA, USA), under N_2 _atmosphere, to avoid oxidation. Acid was removed by vacuum at 40°C. Monosaccharide compositional analysis was accomplished by preparing alditol acetate derivatives of the glycan components, followed by gas chromatography-mass spectrometry (GC/MS) analysis as described [52].

## List of abbreviations

Abs, absorbance; DNA, deoxyribonucleic acid; Dps, DNA binding protein from starved cells; Da, Dalton; ELLA, enzyme-linked lectin assay; ELISA, enzyme-linked immunosorbent assay; ESI-MS, electron-spray ionization mass spectrometry; *O*-GlcNAc, N-acetyl-glucosamine *O*-linked to the protein backbone; PAGE, polyacrylamide gel electrophoresis; PBS, phosphate-buffered saline; SDS, sodium dodecyl sulphate; Std, standard.

## Authors' contributions

ESH and ESB performed the experiments and drafted the manuscript. ESH, MB, MCRB, APC and ESB participated in the study design and coordination. MVS designed and coordinated the *N*-terminal sequencing of J-Dps. ICA designed and performed the ESI-MS and GC-MS analyses. MCRB and MB conceived the project and applied for the grants to fund ESH. All authors read and approved the final manuscript.
